# Uncovering the Mechanism of Aggregation of Human Transthyretin*

**DOI:** 10.1074/jbc.M115.659912

**Published:** 2015-10-12

**Authors:** Lorena Saelices, Lisa M. Johnson, Wilson Y. Liang, Michael R. Sawaya, Duilio Cascio, Piotr Ruchala, Julian Whitelegge, Lin Jiang, Roland Riek, David S. Eisenberg

**Affiliations:** From the ‡ Department of Biological Chemistry, Department of Chemistry and Biochemistry, and Howard Hughes Medical Institute, UCLA, Los Angeles, California 90095-1570,; § Swiss Federal Institute of Technology in Zürich (ETH), Physical Chemistry, ETH Hönggerberg, 8093 Zürich, Switzerland, and; the ¶ Department of Psychiatry and Biobehavioral Sciences, UCLA and The Pasarow Mass Spectrometry Laboratory, The Jane and Terry Semel Institute for Neuroscience and Human Behavior, Los Angeles, California 90024

**Keywords:** amyloid, inhibition mechanism, peptide interaction, protein aggregation, x-ray crystallography, mutational analysis, TTR, transthyretin amyloidosis

## Abstract

The tetrameric thyroxine transport protein transthyretin (TTR) forms amyloid fibrils upon dissociation and monomer unfolding. The aggregation of transthyretin has been reported as the cause of the life-threatening transthyretin amyloidosis. The standard treatment of familial cases of TTR amyloidosis has been liver transplantation. Although aggregation-preventing strategies involving ligands are known, understanding the mechanism of TTR aggregation can lead to additional inhibition approaches. Several models of TTR amyloid fibrils have been proposed, but the segments that drive aggregation of the protein have remained unknown. Here we identify β-strands F and H as necessary for TTR aggregation. Based on the crystal structures of these segments, we designed two non-natural peptide inhibitors that block aggregation. This work provides the first characterization of peptide inhibitors for TTR aggregation, establishing a novel therapeutic strategy.

## Teaser

**Background:** Transthyretin (TTR) aggregation is associated with systemic amyloidosis.

**Results:** Residue replacements on the F and H strands hinder TTR aggregation.

**Conclusion:** The F and H strands are aggregation-driving segments of TTR. The binding of designed peptides inhibits protein aggregation.

**Significance:** We point the way to new therapeutic approaches against TTR aggregation by using peptides to block amyloid segments.

## Introduction

Transthyretin (TTR)^3^ is a 55-kDa protein that aggregates into amyloid fibrils under pathological conditions. In its native tetrameric conformation, TTR transports retinol-binding protein and thyroxine (T_4_) in the blood and cerebrospinal fluid (1). T_4_ binds in a hydrophobic pocket at the center of the tetramer. Amyloid aggregation of TTR occurs by dissociation of tetrameric TTR into monomers; these partially unfold into amyloidogenic intermediates and self-associate into soluble oligomers and amyloid aggregates (2–7). Familial point mutations are known to destabilize the tetramer, leading to faster dissociation and consequent amyloid aggregation (8). Compounds that bind to the hydrophobic T_4_-binding site are known to stabilize the tetramer and slow aggregation (9, 10).

The aggregation of TTR causes transthyretin amyloidosis (ATTR) associated with three conditions traditionally known as senile systemic amyloidosis, familial amyloidotic polyneuropathy, and familial amyloidotic cardiomyopathy. Senile systemic amyloidosis is a late onset disease in which wild-type (WT) TTR aggregates, weakening the heart muscle (2, 11). Senile systemic amyloidosis is usually diagnosed by post-mortem exams of patients over 80 years old. Familial amyloidotic polyneuropathy and familial amyloidotic cardiomyopathy are hereditary conditions characterized by extracellular deposition of TTR amyloid fibrils in the peripheral nerves and heart, respectively, which leads to system failure (12). Familial amyloidotic polyneuropathy is caused by a number of TTR mutations, including L55P and V30M, which we examine here (13). The most common familial amyloidotic cardiomyopathy mutation, V122I, is carried by 3.9% of the African-American population (14, 15).

Currently, there is no cure for transthyretin amyloidosis, and the treatment for familial cases of ATTR is liver transplantation. Tafamidis, a TTR tetramer stabilizer, has been recently approved in Europe; it delays progression of the disease. Several other therapeutics are currently in clinical trials, including other tetramer stabilizers such as diflunisal and RNAi therapies that cause a decrease in the production of TTR protein (16–18). Additional approaches are needed to prevent ATTR, and here we explore the use of peptide inhibitors that block aggregation of TTR.

Several models of the TTR amyloid spine have been proposed (19–22), but the aggregation-prone segments of the protein remain uncertain. Based on the studies of crystal structures of amyloid-driving segments, our group has proposed that fibrils can form through intermolecular self-association of one to several fibril-driving segments. Identical segments from several protein molecules stack into steric zipper structures, which form the spine of the amyloid fibril through tightly interdigitated β-sheets (23–25). Here we identify two segments of TTR that drive protein aggregation by self-association and formation of steric zipper spines of amyloid fibrils. Based on the amyloid structure of these two segments, we designed two peptide inhibitors that halt the progression of TTR aggregation.

## Experimental Procedures

### 

#### 

##### Preparation of Recombinant TTR

Mutants of TTR were produced using a QuikChange site-directed mutagenesis kit (Stratagene). Wild-type and mutated TTR genes were cloned into the pET24(+) vector (Novagen), and the sequences were verified by DNA sequencing and/or later x-ray crystallography of pure proteins (Table 1). For protein expression, exponentially growing *Escherichia coli* Rosetta^TM^(DE3) pLysS competent cells (Millipore) transformed with each of the plasmids were exponentially grown and treated with 1 mm isopropyl β-d-thiogalactoside for 3 h. Wild-type TTR or its variants were purified by nickel affinity chromatography using HisTrap columns (GE Healthcare) following the manufacturer's instructions. Fractions were pooled and subjected to gel filtration chromatography using a HiLoad 16/60 Superdex 75 gel filtration column (GE Healthcare) running on an ÄKTA FPLC system. TTR-rich fractions were confirmed by SDS-PAGE. After checking that the His tag did not modify TTR aggregation (data not shown), we chose to retain the tag to facilitate further analysis of TTR aggregation by His probe blotting.

**TABLE 1:** **Statistics of data collection and atomic refinement** Values in parentheses correspond to the highest resolution shell. The structures of the TTR variants were used to confirm the correct substitutions. The wild-type TTR structure was used for docking. Coordinates are deposited in the Protein Data Bank under the accession codes that are listed in the table. r.m.s.d., root mean square deviation; CC1/2, correlation coefficient.

	WT TTR	AEVVFT	TAVVTN	TTR-V30M	TTR-L55P	TTR-T119Y	TTR-T119W	TTR-S85P	TTR-E92P	TTR-S85P/E92P
**Protein Data Bank code**	**4TLT**	**4XFN**	**4XFO**	**4TL4**	**4TKW**	**4TNE**	**4TM9**	**4TL5**	**4TLS**	**4TLK**
**Data collection**										
Resolution (Å)	50.5-1.7	14.1-1.9	16.0-1.4	51.2-1.8	19.9-1.8	50.9-1.6	8.9-1.7	63.9-1.4	64.4-1.4	63.4-1.4
Space group	P2_1_2_1_2	P2_1_2_1_2_1_	P1	P2_1_2_1_2	P2_1_2_1_2	P2_1_2_1_2	P2_1_2_1_2	P2_1_2_1_2	P2_1_2_1_2	P2_1_2_1_2
Unit cell dimensions (Å)										
*a*	41.24	42.75	4.75	43.06	43.06	42.81	42.71	43.17	42.38	41.98
*b*	84.24	9.53	10.66	85.28	84.40	85.63	85.69	85.8	86.08	85.24
*c*	63.08	18.80	16.39	64.00	65.09	63.22	63.76	63.87	64.36	63.38
Unit cell angles (°)										
α	90.0	90.0	77.6	90.0	90.0	90.0	90.0	90.0	90.0	90.0
β	90.0	90.0	87.8	90.0	90.0	90.0	90.0	90.0	90.0	90.0
γ	90.0	90.0	77.6	90.0	90.0	90.0	90.0	90.0	90.0	90.0
Measured reflections	159,467	2,704	2,662	109,820	142,122	223,096	167,429	232,301	339,017	307,042
Unique reflections	24,512	740	629	24,104	22,582	34,426	26,267	43,105	52,487	48,638
Completeness (%)	98.5 (97.4)	93.5 (95.8)	91.9 (83.1)	98.1 (89.8)	99.4 (98.2)	99.6 (98.0)	98.7 (96.7)	98.7 (88.3)	99.9 (99.6)	99.0 (88.3)
*R*_merge_ (%)*^a^*	6.0 (99.4)	19.7 (46.4)	18.9 (49.6)	7.6 (78.0)	9.8 (123.3)	8.3 (66.6)	5.9 (72.1)	6.2 (82.9)	7.0 (80.5)	6.7 (120.8)
CC_1/2_ (%)	99.9 (70.3)	99.9 (57.3)	99.3 (48.8)	99.9 (67.4)	99.9 (68.1)	99.6 (83.4)	100.0 (79.4)	99.5 (64.6)	99.7 (81.9)	99.5 (54.4)
*I*/σ*I*	18.5 (2.1)	4.7 (2.2)	6.9 (2.7)	12.5 (1.9)	10.8 (1.6)	13.6 (2.8)	21.5 (2.8)	14.8 (1.5)	14.2 (2.4)	13.3 (1.2)

**Refinement**										
Final *R*_work_*^b^*	0.184	0.154	0.134	0.178	0.193	0.168	0.160	0.184	0.172	0.165
Final *R*_free_*^c^*	0.208	0.201	0.148	0.227	0.234	0.198	0.188	0.203	0.195	0.185
r.m.s.d. bond length (Å)	0.007	0.007	0.009	0.017	0.009	0.013	0.011	0.006	0.008	0.009
r.m.s.d. bond angle (°)	1.006	1.054	1.270	1.551	1.145	1.413	1.318	1.054	1.187	1.273
Number of protein atoms	1,784	94	42	1,799	1,725	1,814	1,799	1,794	1,768	1,794
Number of solvent atoms	69	6	0	63	55	101	87	104	111	100
Mean B value (Å^2^)	31.6	9.2	0.8	27.7	37.4	23.3	25.7	24.4	22.1	25.5
Ramachandran torsion angle distributions (%)										
Favored	92.0	100.0	100.0	92.0	90.4	91.5	90.5	90.5	89.9	90.8
Allowed	8.0	0.0	0.0	8.0	9.6	8.5	9.5	9.5	10.1	9.2
Generously allowed	0.0	0.0	0.0	0.0	0.0	0.0	0.0	0.0	0.0	0.0
Disallowed	0.0	0.0	0.0	0.0	0.0	0.0	0.0	0.0	0.0	0.0

*^a^R*_merge_ = Σ*I_j_* − *I*/Σ*I*. Notice that the high R_merge_ value of the highest resolution shell of several structures is compensated by a high CC_1/2_ value.

*^b^R*_work_ = Σ|*F_o_* − *F_c_*|*_j_*/Σ*F_o_*.

*^c^R*_free_ = Σ|*F_o_* − *F_c_*|*_j_*/Σ*F_o_* calculated using a random set containing 5 or 10% of reflections that were not included throughout structure refinement for protein or peptide structure determination, respectively.

##### TTR Aggregation Assay

TTR aggregation assays are described elsewhere (26). Briefly, 1 mg/ml TTR sample in 10 mm sodium acetate (pH 4.3), 100 mm KCl, 10 mm EDTA was incubated at 37 °C for a maximum of 4 days. Various measurements of TTR aggregation were taken. (i) Protein aggregation was monitored by measuring absorbance of the sample at 400 nm. (ii) Protein concentration of the insoluble fraction was calculated as follows. 100 μl of sample was spun at 13,000 rpm for 30 min. The pellet was resuspended in the same volume of fresh buffer and spun again at 13,000 rpm for 30 min. The final pellet was resuspended in 6 m guanidine chloride, and absorbance at 295 nm was measured. Protein concentration of the insoluble fraction was calculated from absorbance data at 295 nm. (iii) TTR aggregation was visualized by transmission electron microscopy of the sample and/or immunodot blot of the insoluble fraction.

Aggregation assays were performed in both the presence and absence of aggregation inhibitors when appropriate at a ratio of 1:10 (TTR monomer:inhibitor) unless labeled otherwise. Turbidity of the sample (absorbance at 400 nm) and protein concentration of the insoluble fraction were measured at time 0, day 1, day 2, and day 4. Values for day 4 are presented in the figures unless labeled otherwise.

##### Immunodot Blot

The aggregation of TTR was followed by dot blot analysis as described (27) using the SuperSignal® West HisProbe^TM^ kit following the manufacturer's instructions (Thermo Scientific). The insoluble fraction of the samples after 4 days of incubation was dotted onto nitrocellulose membranes (0.2 μm; Bio-Rad). A dilution ratio of 1:10,000 was used for the HisProbe antibody.

##### Fibril Formation of TTR Peptides

Peptides were dissolved in PBS buffer (pH 7.4) at a concentration that depended on the solubility of the peptide: ^12^LMVKVL^17^ at 14 mm, ^25^AINVAV^30^ at 17 mm, ^26^NVAVHV^32^ at 16 mm, ^28^VAVHVF^33^ at 15 mm, ^47^GKTSES^52^ at 16 mm, ^65^VEGIYK^70^ at 14 mm, ^68^IYKVEI^73^ at 13 mm, ^80^KALGIS^85^ at 17 mm, ^91^AEVVFT^96^ at 0.5 mm, ^91^APVVFT^96^ at 15.8 mm, ^91^AEVPFT^96^ at 15.8 mm, ^105^YTIAAL^110^ at 15 mm, ^106^TIAALLS^112^ 4.6 mm, and ^119^TAVVTN^124^ at 17 mm. Following dissolution, samples were filtered through a 0.2-μm filter and incubated at 37 °C with no shaking for 2 weeks.

##### Transmission Electron Microscopy (TEM)

TEM was performed to visualize the fibril formation of TTR proteins and peptides. 5 μl of the sample was spotted onto freshly glow-discharged, carbon-coated EM grids (Ted Pella, Redding, CA). After 3 min of incubation, grids were rinsed three times with 5 μl of distilled water and then stained with 2% uranyl acetate for 2 min. Grids were examined with a T12 Quick CryoEM and CryoET (FEI) transmission electron microscope at an accelerating voltage of 120 kV. Digital images were recorded using a Gatan 2,048 × 2,048 charge-coupled device camera.

##### Crystallization and Structure Determination

Crystallization conditions, data collection, and refinement statistics of crystal structures are detailed in Table 1. X-ray diffraction data were collected at the Advanced Photon Source beamline 24-ID-C (for full-length proteins) or 24-ID-E (for peptides). Molecular replacement was performed with the program Phaser (28) using as search models an idealized polyalanine β-strand and chain A of the mutant T119M (Protein Data Bank code 1F86) for peptide and TTR variant data sets, respectively. Crystallographic refinement was performed using PHENIX (29), REFMAC (30), and BUSTER (31). Model building was performed with Coot (32) and illustrated with PyMOL (33).

##### Sequence and Structure Analysis

To predict amyloidogenicity of TTR segments, the TTR sequence was submitted to ZipperDB, the Rosetta-based method that profiles the steric zipper spine, the main structural feature of amyloid fibrils (34, 35). Additionally, solvent-accessible surface area per residue of TTR was calculated to examine the accessibility of the different protein segments for self-association. Solvent-accessible surface area calculations were performed by Areaimol (36) using the structure of WT TTR (Protein Data Bank code 4TLT) in three different conformations: tetramer with 222 symmetry, dimer (the crystallographic asymmetric unit), and monomer (by removing one chain of the dimer). These conformations are based on the dissociation pathway described elsewhere (4).

##### Design of Aggregation Inhibitors

We predicted the interaction of the peptides AEVVFT and TAVVTN with their identical segments at the surface of the monomer or at the tip of a growing steric zipper of TTR based on our finding that the two TTR segments form fibrils in solution by self-aggregation. To validate the prediction, computational docking was carried out using the Rosetta software as described previously (35) using chain A of WT TTR as a template (Protein Data Bank code 4TLT). The optimization of the aggregation inhibitors required the addition of an *N*-methyl group and tetra-arginine tag. The use of the non-natural *N*-methyl groups to protect aggregation inhibitors from proteolysis has been successful in other aggregation inhibitors (37). The addition of a polyarginine tag confers higher solubility and we presume also hinders self-aggregation of the inhibitors. The position of the *N*-methyl group was designed based on our docking model and experimentally checked by aggregation assays.

##### Differential Scanning Calorimetry

Data were taken with a DSC-II differential scanning calorimeter (Calorimetry Sciences Corp., Lindon, UT). The midpoint temperatures of the thermal unfolding transition (*T_m_*) of wild type and mutants were determined. Thermal denaturation of TTR was irreversible under our conditions (1, 38), and absolute thermodynamic parameters were not determined. Previously, researchers found that WT TTR unfolds at 101.7 °C under 2 atm of nitrogen (39). The experiments were performed in 3.5 m guanidinium hydrochloride to depress melting below 100 °C; this guanidinium concentration also solubilized thyroxine. Protein samples were thawed on ice and filtered, and the concentration was measured by UV-visible absorption. Protein samples were diluted to 1 mg/ml in 3.5 m guanidinium chloride, 100 mm sodium acetate (pH 4.3), 100 mm potassium chloride, 1 mm EDTA. A volume of 700 μl of each protein sample was prepared in a 1.5-ml microcentrifuge tube and degassed for 5 min under vacuum while stirring. The reference cell contained only buffer components. Samples were scanned from 25 to 95 °C at a rate of 1 °C/min. The buffer was subtracted, and the baseline was corrected with the linear polynomial function in CPCalc Analysis software. In an additional experiment, T_4_ and aggregation inhibitors were added to WT TTR to determine whether they affect the thermodynamic stability. T_4_ and the aggregation inhibitors were added in 5-fold excess relative to protein concentration to both the reference and sample chambers.

##### Non-denaturing Electrophoresis

Monomeric variant MTTR at 0.5 mg/ml (40) was incubated with increasing concentrations of both TTR inhibitors R4PAm and R4PTm (0–5-fold molar excess) and subjected to blue native electrophoresis using NativePAGE^TM^ Bis-Tris precast gels (4–16% Bis-Tris, 1 mm; Life Technologies) following the manufacturer's instructions. NativeMark^TM^ unstained standards were used for size estimation of proteins.

##### Binding Constant Calculations

The soluble unbound fraction of inhibitors after incubation with the monomeric variant MTTR (40) was quantified by HPLC-MS. Samples were made as follows. Soluble TTR at a fixed concentration of 0.5 mg/ml was incubated at 37 °C for 18 h in 10 mm sodium acetate (pH 7.4), 100 mm KCl, 10 mm EDTA with increasing quantities of combined R4PAm and R4PTm. The aggregated fraction was removed by 0.22-μm filtration. The protein fraction containing soluble TTR and inhibitor-bound complexes was then removed by acid precipitation with 1% acetic acid and consecutive ultracentrifugation at 90,000 rpm for 30 min. Neither 0.22-μm filtration nor acid precipitation affected the concentration of the unbound soluble fraction of the peptide inhibitors (data not shown). The unbound soluble fraction of inhibitors was subjected to size exclusion HPLC followed by mass spectrometry. The instrument used was the Agilent 6460 Triple Quadrupole LC/MS System (Agilent Technologies, Santa Clara, CA) with a TSK gel column (2.0 × 150 mm, 4 μm, 125 Å; Michrom Bioresources, Inc., Auburn, CA). Flow rate was 0.2 ml/min, and the elution buffer was 75% acetonitrile in water containing 0.1% formic acid. Known concentrations of peptide inhibitors were subjected to the same treatment to create a standard curve. The analysis of the results was performed using GraphPad Prism 6.

##### Analytical Size Exclusion Chromatography

To analyze the tetrameric and monomeric populations of TTR, the variant M13R/L17R/T119M (MLT) was generated. This variant forms tetramer and monomers in solution that were examined as follows. A 5-fold molar excess of TTR inhibitors was added to 1 mg/ml soluble MLT and analyzed before and after incubation in 10 mm sodium acetate (pH 4.3), 100 mm KCl, 10 mm EDTA at 37 °C for 18 h. Samples were analyzed by analytical size exclusion with a Superdex 75 16/300 GL column following the manufacturer's instructions (GE Healthcare) running on an ÄKTA FPLC system in 10 mm sodium acetate (pH 7.4), 100 mm KCl, 10 mm EDTA.

## Results

### 

#### 

##### Identifying the Amyloid-driving Segments of TTR

**FIGURE 1.**
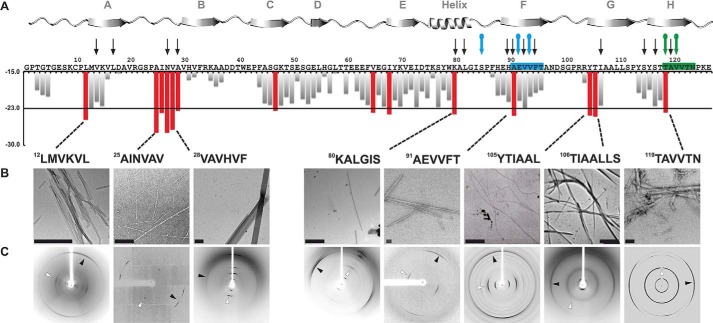
**Identifying amyloidogenic segments in TTR.** *A*, propensities of steric zipper formation of each 6-residue segment within the TTR sequence. Computationally predicted segments with steric zipper propensities are represented with *red bars*. The schematic of the secondary structure of native TTR is shown on *top* of the sequence. *Black arrows* mark residues where proline replacement did not hinder TTR aggregation. *Blue arrows* mark proline replacements that hindered TTR aggregation. The *green arrow* marks the mutation T119M, which protects TTR against fibril formation (58). The *green* and *blue boxes* highlight the segment sequences critical for TTR aggregation that we described in this study. *B*, TEM micrographs of the fibrils formed after 7 days of incubation (*scale bar*, 500 nm). *C*, amyloid x-ray cross-β diffraction pattern of the samples containing fibrils shown in *B*. The *arrowheads* point to the meridional reflection at 4.7–4.8-Å spacings (parallel to the fibril axis; *black arrowheads*) and equatorial reflections at ∼10-Å spacings (*white arrowheads*). Notice that all eight of the examined segments predicted to form amyloid fibrils do in fact form amyloid fibrils.

Our first step toward identifying the amyloid spine-forming segments of TTR was to test candidate segments identified by computational predictions for their ability to aggregate. We used the structure-based, computational method ZipperDB to predict 6-amino acid segments of TTR likely to form amyloid fibrils (34, 35). Of the 14 segments that met the threshold of ZipperDB (Fig. 1*A*, *red bars*), we experimentally tested 12 for their propensity to form fibrils in isolation. Eight of the 12 segments formed amyloid-like aggregates having fibrillar morphology (as viewed by electron microscopy; Fig. 1*B*) and cross-β x-ray diffraction characteristic of amyloid (Fig. 1*C*) (41, 42). The segments that formed fibrils reside in the α-helix and β-strands A, B, F, G, and H of the native structure of TTR. Based on these results, we hypothesized that any of these eight segments could potentially drive TTR aggregation.

**FIGURE 2.**
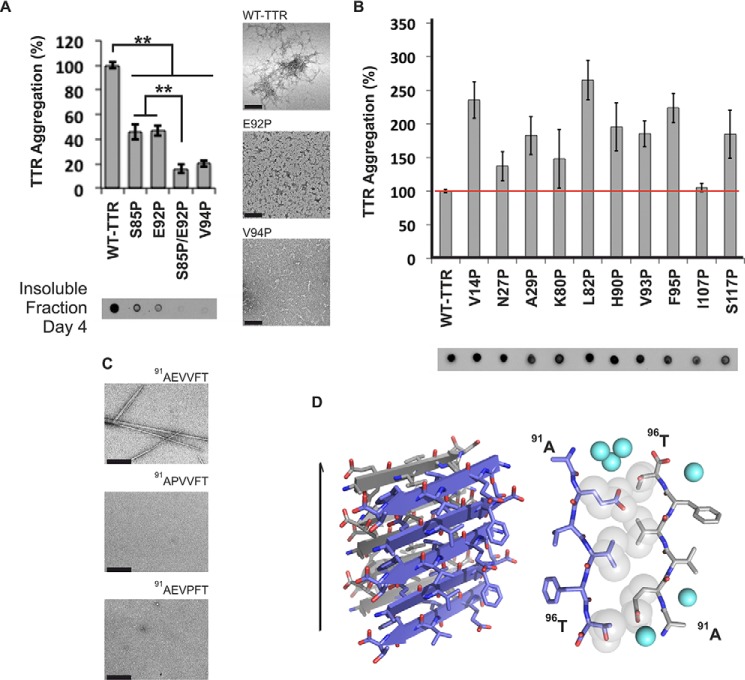
**β-Strand F as an aggregation-driving segment in TTR suitable for aggregation inhibitor design.** TTR aggregation of the protein variants that showed significant delay (*A*) and those that did not decrease protein aggregation (*B*) is shown. Those TTR proline variants that are not shown here were found to be insoluble. Histograms show percentage of TTR aggregation using WT TTR to normalize to 100%. The aggregation was measured by absorbance of the samples at 400 nm after 4 days of incubation at 37 °C and pH 4.3 with no shaking. The *bottom panel* shows a His probe dot blot of the insoluble fraction corresponding to the sample above after solubilization with guanidinium hydrochloride. On the *right* are TEM micrographs of protein aggregates (*scale bar*, 100 nm) after 7 days of incubation. Notice that the proline substitutions within or next to β-strand F hindered TTR aggregation. Several of the other proline substitutions enhanced aggregation; these, like ATTR familial mutations, may alter native structure and/or protein stability (8). *Error bars* represent S.D., and ** symbolizes a *p* value ≤0.003 (*n* = 3). *C*, TEM micrographs of peptides in isolation after 7 days of incubation in PBS with no shaking (*scale bar*, 500 nm). *D*, crystal structure of the segment ^91^AEVVFT^96^ from β-strand F forming a Class-7 steric zipper. One sheet is shown as *blue*; the other is shown as *gray*. On the *left* is a lateral view of the fibril with the fibril axis shown by the *narrow black arrow*. On the *right* is the view down the fibril axis showing two β-sheets in projection. Water molecules are shown as *aquamarine spheres. Spheres* represent the van der Waals radii of the side chain atoms of the tightly packed fibril core.

To pinpoint which of the eight segments drives aggregation within full-length TTR, we created individual proline substitutions within each segment. We chose the substitution to proline because it is known to inhibit fibril formation (43, 44). None of the proline variants fully halted TTR aggregation (Fig. 2, *A* and *B*), but substitutions S85P, E92P, and V94P and the S85P/E92P double substitution significantly decreased protein aggregation and altered aggregate morphology (Fig. 2*A*). In the isolated segment ^91^AEVVFT^96^, proline replacements in positions 92 and 94 completely prevented fibrillization (Fig. 2*C*). These data suggest that the ^91^AEVVFT^96^ segment and the N-terminally adjacent region of full-length TTR are important for fibril formation.

To better understand how ^91^AEVVFT^96^ self-associates to form fibrils, we determined the structure of the fibril-like crystals of ^91^AEVVFT^96^. We found that it forms a Class-7 steric zipper (Ref. 25 and Fig. 2*D*) in which the β-strands stack into antiparallel β-sheets with identical side chains on both faces. This structure shows how ^91^AEVVFT^96^ may self-associate in driving TTR aggregation.

**FIGURE 3.**
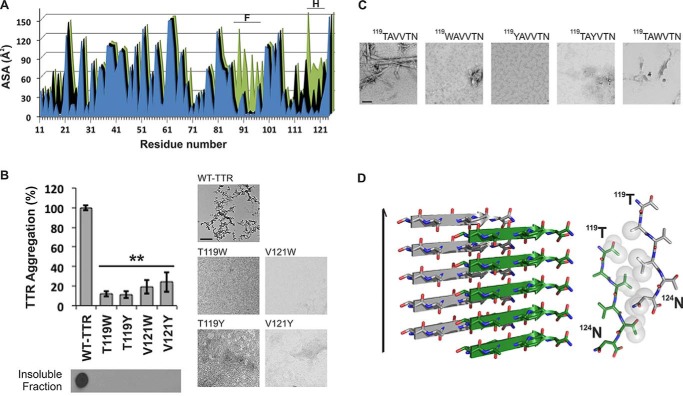
**β-Strand H as a second aggregation-driving segment suitable as a target for aggregation inhibitor design.** *A*, residue solvent-accessible surface area (*ASA*; Å^2^) of TTR was calculated by Areaimol using the structure of WT TTR in three different conformations: tetramer (*blue*), dimer (*black*), and monomer (*green*). Notice that the strands F and H are indeed more exposed when TTR is in a monomeric form. 5.7% of the surface of the amyloidogenic segment ^91^AEVVFT^96^ is solvent-exposed in the tetramer, 5.7% is exposed in the dimer, and 32.1% is exposed in the monomer. Additionally, the amyloidogenic segment ^119^TAVVTN^124^ from strand H is 27.4% solvent-exposed in the tetramer, 43.4% solvent-exposed in the dimer, and 55.3% solvent-exposed in the monomer. *B*, TTR aggregation of variants with substitutions on strand H. The histogram at the *top* shows the percentage of TTR aggregation after 4 days of incubation measured by absorbance at 400 nm with WT aggregation normalized to 100%. *Below*, a His probe dot blot shows the insoluble fraction of the samples after 4 days of incubation and solubilization with guanidinium hydrochloride. *Bottom panels*, TEM images from the samples after 7 days of incubation. These show that the substitutions of residues Thr^119^ and Val^121^ did indeed hinder protein aggregation. *Error bars* represent S.D., and ** symbolizes a *p* value ≤0.003 (*n* = 3). *C*, TEM micrographs of peptides in isolation after 7 days of incubation in PBS with no shaking (*scale bar*, 500 nm). *D*, crystal structure of the segment ^119^TAVVTN^124^ from β-strand H forming a Class-2 steric zipper. One sheet of the zipper is *gray*; the other is *green*. On the *left* is a lateral view of the fibril with the fibril axis indicated by the *narrow black arrow*. On the *right* is the view down the fibril axis showing two β-sheets in projection. *Spheres* represent the van der Waals radii of the side chain atoms of the tightly packed fibril core.

Next, we considered the possibility that TTR contains more than one aggregation-driving segment. Experiments of others show that the fibril core of TTR contains most of the protein, suggesting that more than one segment participates in the fibril core (19, 45). Because the monomeric state is more amyloidogenic than the tetrameric state (4), we reasoned that fibril-driving segments are buried in the tetramer but exposed in the monomer. In this situation, the segments would be exposed to bind to identical segments from other monomers to form a fibril. We analyzed the solvent-accessible surface area of TTR and found that β-strand F is indeed more exposed in the TTR monomer than in the dimer or tetramer (Fig. 3*A*). A second region was also more exposed in the monomer: β-strand H. We therefore tested whether β-strand H might also be a driver of TTR aggregation.

We found that strand H also contributes to TTR amyloid aggregation but not by applying the method of proline substitution that was effective with strand F. Proline substitutions in strand H did not hinder TTR aggregation (Fig. 2). Therefore, we decided to disrupt the capacity for a steric zipper by strand H by substituting residues Thr^119^ and Val^121^ with the bulky residues tyrosine and tryptophan. The size of these side chains is expected to prevent formation of tightly packed steric zippers formed from amino acids with short side chains such as Thr^119^ and Val^121^. Aggregation assays of TTR variants showed that the mutants T119W, T119Y, V121W, and V121Y did not produce aggregates after 4 days of incubation (Fig. 3*B*). For additional evidence, we synthesized and analyzed 6-residue peptides containing the same substitutions, and they did not form aggregates or fibrils in isolation (Fig. 3*C*). These experiments suggest that strand H plays a role during TTR aggregation by acting as a fibril-forming segment.

Next, we performed structural analysis of the isolated strand H segment to test whether it forms a steric zipper structure. We found that ^119^TAVVTN^124^ forms a Class-2 steric zipper (Ref. 25 and Fig. 3*D*) in which the β-strands stack into parallel β-sheets with one sheet face packing against another sheet back. Taken together, these experiments suggest that the ^119^TAVVTN^124^ segment is important for fibril formation.

##### Validation of the Role of Strands F and H in Aggregation by the Design of Amyloid Inhibitors

**FIGURE 4.**
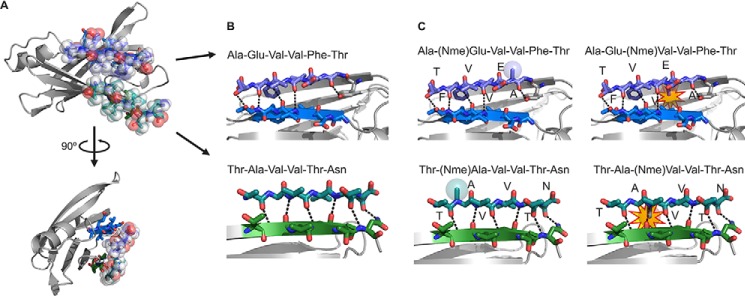
**Design of sequence-specific peptide inhibitors of TTR aggregation.** *A*, computational docking model of the peptides AEVVFT and TAVVTN bound to strands F (*blue*) and H (*green*), respectively, of the TTR monomer. Peptides are shown with *translucent spheres* representing van der Waals radii; the TTR monomer is shown as *ribbons* with side chains of strands F and H as *sticks. B*, lateral, close-up view of the F and H strands in the docking model showing the designed pattern of hydrogen bonding (*dashed lines*) between the peptides (*sticks*) and the TTR β-strands (*ribbon* with *sticks*). *C*, docking models of *N*-methylated peptides predict which will bind TTR (same view as *B*). *N*-Methylations were added to increase peptide solubility, reduce aggregation, and reduce sensitivity. *Colored spheres* represent the van der Waals radii of the *N*-methyl groups (*Nme*) in favorable positions; *yellow stars* highlight clashes of the *N*-methyl modifications with the TTR monomer.

To confirm that strands F and H drive protein aggregation, we tested aggregation inhibition by short peptides that we designed to bind to strands F and H, thereby shielding them from aggregation. We designed amyloid inhibitors against β-strands F and H using prior strategies that have been successful at blocking aggregation of other proteins (46, 47). The design was supported by protein-protein docking (Fig. 4). To create aggregation inhibitors capable of binding the target sequence without self-associating, we modified the sequences ^91^AEVVFT^96^ and ^119^TAVVTN^124^ by adding non-natural amino acids and a charged tag. We used the docking model to predict the most suitable positions for *N*-methylated residues. The model predicted that even positions (Glu^92^, Val^94^, and Thr^96^ from ^91^AEVVFT^96^ and Ala^120^, Val^122^, and Asn^124^ from ^119^TAVVTN^124^) were more favorable than odd positions (Fig. 4*C*). This prediction was later supported by experiments.

**FIGURE 5.**
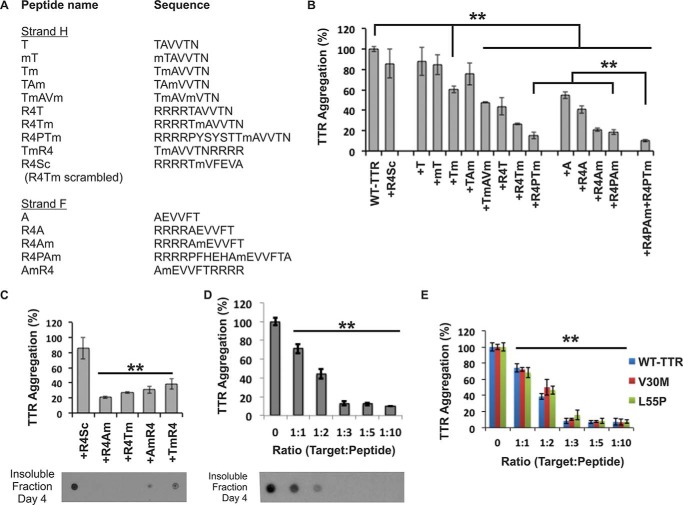
**Designed peptides are sequence-specific inhibitors of TTR aggregation.** *A*, list of inhibiting peptides tested as TTR aggregation inhibitors. *B–E*, evaluation of the peptide inhibitors. The graphs show the percentage of TTR aggregation after 4 days of incubation in the presence or absence of peptide inhibitor measured by absorbance at 400 nm; WT inhibition-free aggregation was normalized to 100%. The initial concentration of soluble TTR was 1 (*B–D*) or 0.2 mg/ml, which corresponds to the concentration of TTR in plasma (*E*). The molar excess of peptide inhibitor over target (TTR monomer) is 3-fold unless labeled otherwise. In *C* and *D*, a His probe dot blot shows the insoluble fraction of the samples after 4 days of incubation and solubilization with guanidinium hydrochloride. *B*, initial screening of inhibitors showing that three modifications significantly improved the effectiveness: (i) increasing the length of the matched sequence of the peptide and the target, (ii) addition of a charged tag, and (iii) addition of an *N*-methyl group in positions predicted to be favorable by the docking model (Fig. 4*C*). The two best inhibitors of TTR aggregation were R4PAm and R4PTm. *C*, the peptide inhibitors are more effective with the charged tag at the N terminus than at the C terminus. *D*, dose-dependent effectiveness of the peptides R4PAm and R4PTm in combination. Maximal inhibition of TTR aggregation is reached when the target to peptide molar ratio is 1:3. *E*, aggregation assay of the familial mutants V30M and L55P in the presence of R4PAm and R4PTm in combination using a physiological concentration of protein (3.6 μm). *Error bars* represent S.D. (*n* = 3), and ** symbolizes a *p* value ≤0.003.

Fifteen different peptides were tested as potential inhibitors of aggregation (Fig. 5). The peptide sequences are listed in Fig. 5*A*. We began by testing the 6-residue F- and H-strand peptides, AEVVFT and TAVVTN, with sequential modifications of *N*-methylated amino acids (Fig. 5*B*). The experimental observation that the aggregation inhibitors with *N*-methyl residues at even positions were more effective than those with *N*-methylated odd positions agrees with our docking model (Figs. 4*C* and 5*B*). We next added an N-terminal tag of four arginine residues to further improve the inhibitors by increasing their solubility and preventing self-aggregation. The addition of the N-terminal residues significantly increased the inhibition (Fig. 5*B*). The position of the tag was also tested at both termini, and we found that the N-terminally tagged peptides were better inhibitors (Fig. 5*C*).

We assessed the specificity of the inhibitors in two ways. First, we tested a scrambled control peptide with the polyarginine tag intact (Fig. 5*B*). Charged tags might form salt bridges with several of the loops in TTR that are rich in acidic residues (48). The control peptide did not disrupt protein aggregation, confirming that the aggregation inhibition is sequence-specific and not reliant on the arginine tag (Fig. 5*B*). Then we tested the combination of F and H inhibitors. The peptides R4PAm and R4PTm showed a synergistic effect, suggesting two different binding sites (Fig. 5*B*), in a dose-dependent manner (Fig. 5*D*). The combination was also effective at blocking aggregation of the familial mutants V30M and L55P at physiological TTR concentration (Fig. 5*E*). Based on this initial screening, the two peptides R4PAm and R4PTm in combination were selected for further study.

**FIGURE 6.**
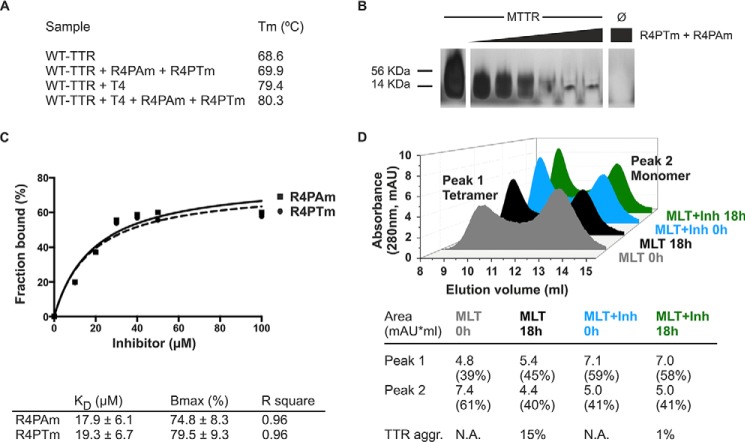
**Analysis of the mechanism of action of the TTR inhibitors.** *A*, midpoint temperatures of the thermal unfolding transition (*T_m_*) of wild-type TTR at different conditions were determined by differential scanning calorimetry. Relative stability is compared in the presence and absence of a 5:1 molar ratio of T_4_ and/or the combination of R4PAm and R4PTm to TTR monomer. The TTR concentration was 1 mg/ml. Notice that the addition of the natural ligand T_4_ increased the protein thermostability by 10.8 °C. The addition of the peptide inhibitors increased it only by 1.3 °C in the absence of T_4_ and by 0.9 °C when the ligand was present. *B*, inhibition of oligomer formation of MTTR after incubation with R4PTm and R4PAm. A fixed amount of MTTR (0.5 mg/ml) was incubated in the presence of increasing concentrations of TTR inhibitors (0–5-fold excess) and subjected to non-denaturing electrophoresis. The sizes of His-tagged TTR monomer (14 kDa) and tetramer (56 kDa) are shown next to the gel. R4PTm and R4PAm inhibited MTTR oligomer formation in a dose-dependent manner. *C*, fraction of the inhibitors R4PAm (*solid line*, *squares*) and R4PTm (*dashed line*, *circles*) bound to MTTR. The soluble fraction of the samples was collected after ultracentrifugation followed by acid precipitation of soluble MTTR and inhibitor-bound complexes. The unbound fraction of inhibitors was analyzed by HPLC-MS. One-site specific binding analysis was performed using GraphPad Prism 6 to calculate binding parameters *K_D_* as apparent binding affinity and *B*_max_ as maximum bound fraction. *D*, analysis of tetrameric and monomeric populations of MLT after incubation with TTR inhibitors. Size exclusion chromatography of MLT reveals that the presence of TTR inhibitors does not drive the protein equilibrium to populate the monomeric species. In contrast, an increase of tetrameric species was found after incubation with inhibitors (*Inh*). TTR aggregation (*TTR agg.*) was measured by absorbance at 400 nm with the initial protein amount normalized to 100%. *mAU*, milli-absorbance units; *N.A.*, not applicable.

To further characterize the mechanism of action of the inhibitor pair, we examined their effect on different assembly states of TTR. First, we examined tetramer stability by differential scanning calorimetry (Fig. 6*A*). We found that the inhibitors did not affect WT TTR stability or compete with T_4_ for its binding site (Fig. 6*A*). These results confirm that the mechanism for inhibiting aggregation is different from tetramer stabilization (9, 10, 49). Additionally, we examined the effect on oligomer formation of an engineered monomeric variant of TTR, MTTR (40). MTTR has two methionine substitutions at residues Phe^87^ and Leu^110^, which prevent dimer and tetramer formation, leading to rapid aggregation at acidic pH. Incubation of MTTR with the inhibitor pair reduced oligomer formation as monitored by non-denaturing gel electrophoresis (Fig. 6*B*). The analysis of the binding of the TTR inhibitors to MTTR species showed an apparent binding constant of 17.9 ± 6.1 and 19.3 ± 6.7 μm for R4PAm and R4PTm, respectively (Fig. 6*C*). This experiment did not exclude the binding of the inhibitors to aggregated species. The measured affinity is therefore a combined affinity for all species in solution, and the monomer-specific affinity might be weaker than calculated.

We next found that the presence of inhibitors leads to an equilibrium shift toward the tetramer population. We first generated the TTR variant MLT, which exhibits monomers and tetramers in solution, and we analyzed both populations by size exclusion chromatography (Fig. 6*D*). The results show that the addition of inhibitors promotes an immediate increase in tetramer concentration when compared with the absence of inhibitors. Additionally, the monomeric concentration remains unchanged after incubation for 18 h in the presence of the inhibitors, whereas their absence results in monomer loss and aggregation. Taken together, these results suggest a mechanism of action that does not stabilize the monomer population and differs from the tetramer stabilization.

In summary, our results indicate that strands F and H play a causative role in TTR aggregation. We used this hypothesis to design inhibitors of their self-aggregation to stop amyloid formation of wild-type TTR and familial mutant variants.

## Discussion

TTR aggregation is linked to ATTR, a condition that is not yet curable (50). Although new strategies are being evaluated (16–18), the development of combinatory systems that address various molecular processes driving TTR aggregation is desirable to maximize treatment options. Here we propose a novel mechanism of aggregation inhibition that consists of targeting segments of TTR that are exposed only when the TTR tetramer dissociates and drive protein aggregation.

Others have shown that a necessary step in the conversion of TTR to amyloid fibrils is its dissociation to a monomer (2–7), but the specific segments that cause the aggregation of monomers had not previously been identified. We performed experiments to identify adhesive segments in TTR that participate in fibril formation. Our procedure was first to identify possible amyloid-forming segments by ZipperDB three-dimensional profiling. In this procedure, the TTR sequence is computationally threaded through a “profile” derived from the structure of a known amyloid fibril (34, 35). This procedure identified 14 steric zipper-forming 6-residue-long segments, eight of which indeed formed fibrils in solution (Fig. 1). Also our computations with ZipperDB show that TTR familial mutations do not increase the propensity of fibril formation (data not shown). These results support the hypothesis that familial point mutations destabilize the quaternary structure rather than creating new amyloidogenic segments (8). With systematic mutagenesis of the eight fibril-forming segments and computational prediction of exposed strands in the monomer, we were able to trace the cause of TTR fibril formation to the sequence segments that form the F and H β-strands of the native structure.

Mutational analysis of amyloid proteins for identification of aggregation-driving segments has been reported by our group and others to be effective (43, 51, 52). These data led us to strands F and H in the case of TTR (Figs. 2 and 3). However, mutation to proline, tryptophan, or tyrosine does not necessarily imply prevention from aggregation. As additional evidence, we tested fibrillization of the segments ^91^AEVVFT^96^ and ^119^TAVVTN^124^ in isolation as well as their mutated versions to find that the substitution of residue 85, 92, 94, 119, or 121 did hinder self-association and fibril formation (Figs. 2 and 3). In addition, to further prove that β-strands F and H can participate in amyloid core formation of TTR fibrils, we determined the crystal structure of the steric zippers formed by the segments in isolation by x-ray crystallography (23–25, 53). The crystal structure of ^91^AEVVFT^96^ revealed a Class-7 steric zipper with antiparallel β-strands and antiparallel sheets (Ref. 25 and Fig. 2*D*). The side chain organization explains why the residue replacements E92P and V94P hinder TTR aggregation because the proline substitution would decrease the shape complementarity between sheets in the steric zipper structure. Moreover, the crystal structure of ^119^TAVVTN^124^ is a Class-2 steric zipper, face-to-back arrangement with parallel β-strands and parallel sheets (Ref. 25 and Fig. 3*D*). The side chain organization in this peptide also explains why the residue replacements T119W, T119Y, V121W, and V121Y impede TTR aggregation. Because residues Thr^119^ and Val^121^ are pointing to the hydrophobic T_4_-binding site, we cannot dismiss the possibility that the tryptophan or tyrosine could strengthen the hydrophobic contacts to increase the stability of the protein and delay aggregation. However, the involvement of these segments in the formation of TTR fibrils was supported by two pieces of evidence. First, peptides with Tyr/Trp substitutions did not form fibrils in isolation (Figs. 2*C* and 3*C*). Second, the aggregation inhibitors targeting these segments hindered TTR aggregation (Figs. 5 and 6). We do not dismiss the contribution of other aggregation-prone segments to the amyloid core. Strands F and H that we propose here might be segments most susceptible to structural changes in promoting aggregation.

Combining mutational and structural studies of TTR variants and isolated peptides, we uncovered the involvement of the strands F and H in TTR aggregation (Figs. 1–3). This finding is consistent with NMR relaxation dispersion studies that showed that, at physiological pH, monomeric TTR undergoes conformational fluctuations of the H and F strands that are propagated along the entire sheet (54). Kelly and co-workers (48, 54) have also analyzed TTR by NMR studies and x-ray crystallography in neutral and acidic environments to assess structural changes. These studies have confirmed that the EF-loop undergoes large conformational changes when the pH is changed from neutral to an acidic environment. We speculate that the S85P substitution, near strand F, constrains this flexibility to prevent aggregation. Substitutions in strand F, E92P and V94P, most likely hinder self-recognition and further aggregation. Kelly and co-workers (40) have engineered a monomeric variant of TTR that is non-amyloidogenic unless partially denatured. The monomeric variant aggregates much faster than the wild-type tetramer at pH 4.4 (minutes *versus* hours, respectively). They also determined the structure of this variant and showed that most of the deviations from the structure of WT TTR are observed on the interfaces between the subunits, involving strands F and H, which supports our findings. We reasoned that if strands F and H are important for the self-association of TTR then one could stop protein aggregation by blocking the two segments.

To validate our identification of the fibril-forming segments, we designed analog peptides that interact with the F and H strands. These inhibitors slowed fibril formation, supporting our identification of the fibril-forming segments (Figs. 5 and 6). Eisenberg and co-workers (46, 47) have previously used structure-based design of peptides or compounds to disrupt fibril development and/or growth. For the design of TTR aggregation inhibitors, *N*-methyl residues were incorporated into the peptide sequence to increase effectiveness (Figs. 4 and 5*B*). This is advantageous because non-natural amino acids can increase peptide stability by reducing proteolytic degradation (37, 55). This initial screening resulted in two optimized TTR peptide inhibitors, both 16 residues long, with separate binding sites that differ from the hydrophobic pocket (Figs. 5*B* and 6*A*).

The inhibition of TTR aggregation by peptide inhibitors as a prospective therapeutic strategy needs further optimization to improve affinity. Although similar to some amyloid inhibitors (56, 57), the apparent affinity of the monomeric variant MTTR (40) to the inhibitors is low compared with those found for tetramer stabilizers (for instance, see Refs. 9 and 10). However, the binding of the inhibitors to TTR after tetramer dissociation might differ from their binding to MTTR.

The inhibition of TTR aggregation by blocking strands F and H indicates their importance for protein aggregation. Our data suggest that the aggregation of TTR requires tetramer dissociation into monomeric species as suggested elsewhere (2–6). Several other studies propose that TTR aggregation occurs by dimer arrangement (19–21). However, strands F and H are buried in the dimer interface (Ref. 4 and Fig. 3*A*), impeding self-association. Therefore, the inhibition of TTR aggregation by interaction of peptide inhibitors with the F and H strands requires dimer dissociation and segment exposure.

**FIGURE 7.**
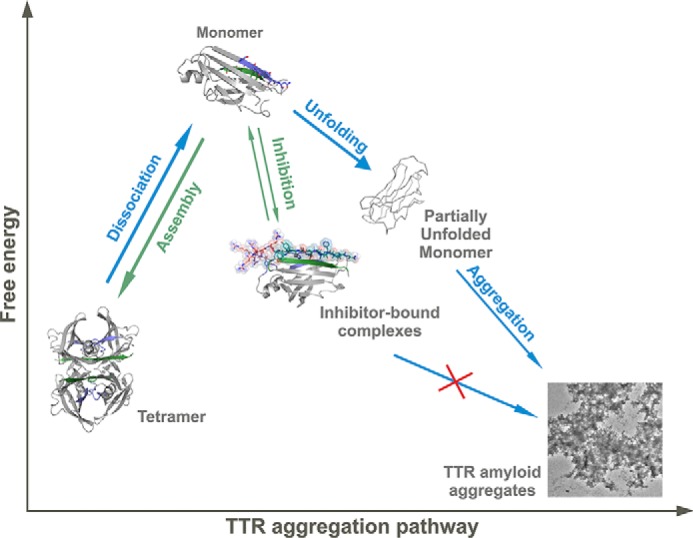
**Model of peptide-based inhibition of TTR aggregation.** The low free energy of TTR amyloid fibrils drives aggregation with TTR dissociation providing a kinetic barrier (4). The inhibitors do not affect stability of the TTR tetramer but bind to intermediate species, hindering unfolding and aggregation. Although the inhibitor-bound complex is more stable than the free monomer, it is less stable than the tetramer, thereby favoring tetramer reassembly. Note that in this scheme the hydrophobic pocket of the tetramer remains accessible for complementary treatment with a stabilizer compound such as tafamidis or diflunisal (10, 49).

Based on our data, we propose a pathway for peptide-based inhibition of TTR aggregation (Fig. 7). Upon tetramer dissociation, the binding of the peptide inhibitors to their identical segments hinders self-recognition and aggregation. The inhibitor-bound TTR complex is more stable than the monomer but less stable than the tetramer (Fig. 6*D*), thereby favoring tetramer reassembly. Additionally, although the inhibitors were designed to interact with the F and H strands after dissociation of the tetramer, they may act at the amyloid level by capping the emerging fibrils to hinder further growth (46). In either case, the steric zipper-like interaction of the peptide inhibitors with the F and H strands would block self-association and protein aggregation.

In summary, we have shown that strands F and H are actively involved in TTR aggregation and uncovered a new strategy for the inhibition of TTR aggregation by small non-natural peptides. Their mechanism of action allows combinatory treatment with tetramer-stabilizing compounds, which might be an effective therapeutic approach. The two inhibiting peptides may serve as leads for the development of drugs against TTR systemic diseases.

## Author Contributions

L. S. and D. S. E. designed the project. L. S., L. M. J., and W. Y. L. performed the research. L. S., L. M. J., and D. S. E. analyzed the data. P. R., J. W., L. J., and R. R. contributed with analytic tools and discussion. D. C. and M. R. S. helped with crystallography and structural analysis. L. S. wrote the paper. Critical revision was done by L. M. J. and D. S. E. All authors reviewed the results and approved the final version of the manuscript.

## Footnotes

3 The abbreviations used are:

TTR: transthyretin
ATTR: transthyretin amyloidosis
TEM: transmission electron microscopy
T_4_: thyroxine
*K_D_*: apparent binding constant
*B*_max_: maximum bound fraction
MTTR: monomeric transthyretin
Bis-Tris: 2-[bis(2-hydroxyethyl)amino]-2-(hydroxymethyl)propane-1,3-diol
MLT: M13R/L17R/T119M.

## References

[1] Chung C. M., Connors L. H., Benson M. D., and Walsh M. T. (2001) Biophysical analysis of normal transthyretin: implications for fibril formation in senile systemic amyloidosis. Amyloid 8, 75–83 11409037 10.3109/13506120109007348

[2] Westermark P., Sletten K., Johansson B., and Cornwell G. G. 3rd (1990) Fibril in senile systemic amyloidosis is derived from normal transthyretin. Proc. Natl. Acad. Sci. U.S.A. 87, 2843–2845 2320592 10.1073/pnas.87.7.2843 PMC53787

[3] Colon W., and Kelly J. W. (1992) Partial denaturation of transthyretin is sufficient for amyloid fibril formation *in vitro*. Biochemistry 31, 8654–8660 1390650 10.1021/bi00151a036

[4] Foss T. R., Wiseman R. L., and Kelly J. W. (2005) The pathway by which the tetrameric protein transthyretin dissociates. Biochemistry 44, 15525–15533 16300401 10.1021/bi051608t

[5] Lai Z., Colón W., and Kelly J. W. (1996) The acid-mediated denaturation pathway of transthyretin yields a conformational intermediate that can self-assemble into amyloid. Biochemistry 35, 6470–6482 8639594 10.1021/bi952501g

[6] Quintas A., Vaz D. C., Cardoso I., Saraiva M. J., and Brito R. M. (2001) Tetramer dissociation and monomer partial unfolding precedes protofibril formation in amyloidogenic transthyretin variants. J. Biol. Chem. 276, 27207–27213 11306576 10.1074/jbc.M101024200

[7] Reixach N., Deechongkit S., Jiang X., Kelly J. W., and Buxbaum J. N. (2004) Tissue damage in the amyloidoses: transthyretin monomers and nonnative oligomers are the major cytotoxic species in tissue culture. Proc. Natl. Acad. Sci. U.S.A. 101, 2817–2822 14981241 10.1073/pnas.0400062101 PMC365703

[8] Hurshman Babbes A. R., Powers E. T., and Kelly J. W. (2008) Quantification of the thermodynamically linked quaternary and tertiary structural stabilities of transthyretin and its disease-associated variants: the relationship between stability and amyloidosis. Biochemistry 47, 6969–6984 18537267 10.1021/bi800636q PMC2667099

[9] Munro S. L., Lim C. F., Hall J. G., Barlow J. W., Craik D. J., Topliss D. J., and Stockigt J. R. (1989) Drug competition for thyroxine binding to transthyretin (prealbumin): comparison with effects on thyroxine-binding globulin. J. Clin. Endocrinol. Metab. 68, 1141–1147 2498384 10.1210/jcem-68-6-1141

[10] Alhamadsheh M. M., Connelly S., Cho A., Reixach N., Powers E. T., Pan D. W., Wilson I. A., Kelly J. W., and Graef I. A. (2011) Potent kinetic stabilizers that prevent transthyretin-mediated cardiomyocyte proteotoxicity. Sci. Transl. Med. 3, 97ra81 10.1126/scitranslmed.3002473 PMC3227540 21865539

[11] Cornwell G. G. 3rd, Murdoch W. L., Kyle R. A., Westermark P., and Pitkänen P. (1983) Frequency and distribution of senile cardiovascular amyloid. A clinicopathologic correlation. Am. J. Med. 75, 618–623 6624768 10.1016/0002-9343(83)90443-6

[12] Misumi Y., Ando Y., Gonçalves N. P., and Saraiva M. J. (2013) Fibroblasts endocytose and degrade transthyretin aggregates in transthyretin-related amyloidosis. Lab. Invest. 93, 911–920 23817086 10.1038/labinvest.2013.83

[13] Sipe J. D. (1994) Amyloidosis. Crit. Rev. Clin. Lab. Sci. 31, 325–354 7888076 10.3109/10408369409084679

[14] Jacobson D. R., Pastore R. D., Yaghoubian R., Kane I., Gallo G., Buck F. S., and Buxbaum J. N. (1997) Variant-sequence transthyretin (isoleucine 122) in late-onset cardiac amyloidosis in black Americans. N. Engl. J. Med. 336, 466–473 9017939 10.1056/NEJM199702133360703

[15] Jiang X., Buxbaum J. N., and Kelly J. W. (2001) The V122I cardiomyopathy variant of transthyretin increases the velocity of rate-limiting tetramer dissociation, resulting in accelerated amyloidosis. Proc. Natl. Acad. Sci. U.S.A. 98, 14943–14948 11752443 10.1073/pnas.261419998 PMC64963

[16] Coelho T., Maia L. F., Martins da Silva A., Waddington Cruz M., Planté-Bordeneuve V., Lozeron P., Suhr O. B., Campistol J. M., Conceição I. M., Schmidt H. H., Trigo P., Kelly J. W., Labaudinière R., Chan J., Packman J., Wilson A., and Grogan D. R. (2012) Tafamidis for transthyretin familial amyloid polyneuropathy: a randomized, controlled trial. Neurology 79, 785–792 22843282 10.1212/WNL.0b013e3182661eb1 PMC4098875

[17] Castaño A., Helmke S., Alvarez J., Delisle S., and Maurer M. S. (2012) Diflunisal for ATTR cardiac amyloidosis. Congest. Heart Fail. 18, 315–319 22747647 10.1111/j.1751-7133.2012.00303.x PMC3727153

[18] Coelho T., Adams D., Silva A., Lozeron P., Hawkins P. N., Mant T., Perez J., Chiesa J., Warrington S., Tranter E., Munisamy M., Falzone R., Harrop J., Cehelsky J., Bettencourt B. R., Geissler M., Butler J. S., Sehgal A., Meyers R. E., Chen Q., Borland T., Hutabarat R. M., Clausen V. A., Alvarez R., Fitzgerald K., Gamba-Vitalo C., Nochur S. V., Vaishnaw A. K., Sah D. W., Gollob J. A., and Suhr O. B. (2013) Safety and efficacy of RNAi therapy for transthyretin amyloidosis. N. Engl. J. Med. 369, 819–829 23984729 10.1056/NEJMoa1208760

[19] Schormann N., Murrell J. R., and Benson M. D. (1998) Tertiary structures of amyloidogenic and non-amyloidogenic transthyretin variants: new model for amyloid fibril formation. Amyloid 5, 175–187 9818054 10.3109/13506129809003843

[20] Serag A. A., Altenbach C., Gingery M., Hubbell W. L., and Yeates T. O. (2002) Arrangement of subunits and ordering of β-strands in an amyloid sheet. Nat. Struct. Biol. 9, 734–739 12219081 10.1038/nsb838

[21] Laidman J., Forse G. J., and Yeates T. O. (2006) Conformational change and assembly through edge β strands in transthyretin and other amyloid proteins. Acc. Chem. Res. 39, 576–583 16981673 10.1021/ar050017s

[22] Pires R. H., Karsai Á., Saraiva M. J., Damas A. M., and Kellermayer M. S. (2012) Distinct annular oligomers captured along the assembly and disassembly pathways of transthyretin amyloid protofibrils. PLoS One 7, e44992 22984597 10.1371/journal.pone.0044992 PMC3440338

[23] Nelson R., Sawaya M. R., Balbirnie M., Madsen A. Ø., Riekel C., Grothe R., and Eisenberg D. (2005) Structure of the cross-β spine of amyloid-like fibrils. Nature 435, 773–778 15944695 10.1038/nature03680 PMC1479801

[24] Nelson R., and Eisenberg D. (2006) Structural models of amyloid-like fibrils. Adv. Protein Chem. 73, 235–282 17190616 10.1016/S0065-3233(06)73008-X

[25] Sawaya M. R., Sambashivan S., Nelson R., Ivanova M. I., Sievers S. A., Apostol M. I., Thompson M. J., Balbirnie M., Wiltzius J. J., McFarlane H. T., Madsen A. Ø., Riekel C., and Eisenberg D. (2007) Atomic structures of amyloid cross-β spines reveal varied steric zippers. Nature 447, 453–457 17468747 10.1038/nature05695

[26] Hurshman A. R., White J. T., Powers E. T., and Kelly J. W. (2004) Transthyretin aggregation under partially denaturing conditions is a downhill polymerization. Biochemistry 43, 7365–7381 15182180 10.1021/bi049621l

[27] Kayed R., Head E., Sarsoza F., Saing T., Cotman C. W., Necula M., Margol L., Wu J., Breydo L., Thompson J. L., Rasool S., Gurlo T., Butler P., and Glabe C. G. (2007) Fibril specific, conformation dependent antibodies recognize a generic epitope common to amyloid fibrils and fibrillar oligomers that is absent in prefibrillar oligomers. Mol. Neurodegener. 2, 18 17897471 10.1186/1750-1326-2-18 PMC2100048

[28] McCoy A. J., Grosse-Kunstleve R. W., Adams P. D., Winn M. D., Storoni L. C., and Read R. J. (2007) Phaser crystallographic software. J. Appl. Crystallogr. 40, 658–674 19461840 10.1107/S0021889807021206 PMC2483472

[29] Zwart P. H., Afonine P. V., Grosse-Kunstleve R. W., Hung L. W., Ioerger T. R., McCoy A. J., McKee E., Moriarty N. W., Read R. J., Sacchettini J. C., Sauter N. K., Storoni L. C., Terwilliger T. C., and Adams P. D. (2008) Automated structure solution with the PHENIX suite. Methods Mol. Biol. 426, 419–435 18542881 10.1007/978-1-60327-058-8_28

[30] Murshudov G. N., Vagin A. A., and Dodson E. J. (1997) Refinement of macromolecular structures by the maximum-likelihood method. Acta Crystallogr. D Biol. Crystallogr. 53, 240–255 15299926 10.1107/S0907444996012255

[31] Blanc E., Roversi P., Vonrhein C., Flensburg C., Lea S. M., and Bricogne G. (2004) Refinement of severely incomplete structures with maximum likelihood in BUSTER-TNT. Acta Crystallogr. D Biol. Crystallogr. 60, 2210–2221 15572774 10.1107/S0907444904016427

[32] Emsley P., and Cowtan K. (2004) Coot: model-building tools for molecular graphics. Acta Crystallogr. D Biol. Crystallogr. 60, 2126–2132 15572765 10.1107/S0907444904019158

[33] DeLano W. L. (2010) The PyMOL Molecular Graphics System, Schrödinger, LLC, New York

[34] Thompson M. J., Sievers S. A., Karanicolas J., Ivanova M. I., Baker D., and Eisenberg D. (2006) The 3D profile method for identifying fibril-forming segments of proteins. Proc. Natl. Acad. Sci. U.S.A. 103, 4074–4078 16537487 10.1073/pnas.0511295103 PMC1449648

[35] Goldschmidt L., Teng P. K., Riek R., and Eisenberg D. (2010) Identifying the amylome, proteins capable of forming amyloid-like fibrils. Proc. Natl. Acad. Sci. U.S.A. 107, 3487–3492 20133726 10.1073/pnas.0915166107 PMC2840437

[36] Winn M. D., Ballard C. C., Cowtan K. D., Dodson E. J., Emsley P., Evans P. R., Keegan R. M., Krissinel E. B., Leslie A. G., McCoy A., McNicholas S. J., Murshudov G. N., Pannu N. S., Potterton E. A., Powell H. R., Read R. J., Vagin A., and Wilson K. S. (2011) Overview of the CCP4 suite and current developments. Acta Crystallogr. D Biol. Crystallogr. 67, 235–242 21460441 10.1107/S0907444910045749 PMC3069738

[37] Cruz M., Tusell J. M., Grillo-Bosch D., Albericio F., Serratosa J., Rabanal F., and Giralt E. (2004) Inhibition of β-amyloid toxicity by short peptides containing N-methyl amino acids. J. Pept. Res. 63, 324–328 15049845 10.1111/j.1399-3011.2004.00156.x

[38] Takeuchi M., Mizuguchi M., Kouno T., Shinohara Y., Aizawa T., Demura M., Mori Y., Shinoda H., and Kawano K. (2007) Destabilization of transthyretin by pathogenic mutations in the DE loop. Proteins 66, 716–725 17143887 10.1002/prot.21252

[39] Shnyrov V. L., Villar E., Zhadan G. G., Sanchez-Ruiz J. M., Quintas A., Saraiva M. J., and Brito R. M. (2000) Comparative calorimetric study of non-amyloidogenic and amyloidogenic variants of the homotetrameric protein transthyretin. Biophys. Chem. 88, 61–67 11152276 10.1016/s0301-4622(00)00199-x

[40] Jiang X., Smith C. S., Petrassi H. M., Hammarström P., White J. T., Sacchettini J. C., and Kelly J. W. (2001) An engineered transthyretin monomer that is nonamyloidogenic, unless it is partially denatured. Biochemistry 40, 11442–11452 11560492 10.1021/bi011194d

[41] Jahn T. R., Makin O. S., Morris K. L., Marshall K. E., Tian P., Sikorski P., and Serpell L. C. (2010) The common architecture of cross-β amyloid. J. Mol. Biol. 395, 717–727 19781557 10.1016/j.jmb.2009.09.039

[42] Eisenberg D., and Jucker M. (2012) The amyloid state of proteins in human diseases. Cell 148, 1188–1203 22424229 10.1016/j.cell.2012.02.022 PMC3353745

[43] Ivanova M. I., Sievers S. A., Guenther E. L., Johnson L. M., Winkler D. D., Galaleldeen A., Sawaya M. R., Hart P. J., and Eisenberg D. S. (2014) Aggregation-triggering segments of SOD1 fibril formation support a common pathway for familial and sporadic ALS. Proc. Natl. Acad. Sci. U.S.A. 111, 197–201 24344300 10.1073/pnas.1320786110 PMC3890817

[44] Soto C., Kascsak R. J., Saborío G. P., Aucouturier P., Wisniewski T., Prelli F., Kascsak R., Mendez E., Harris D. A., Ironside J., Tagliavini F., Carp R. I., and Frangione B. (2000) Reversion of prion protein conformational changes by synthetic β-sheet breaker peptides. Lancet 355, 192–197 10675119 10.1016/s0140-6736(99)11419-3

[45] Bergström J., Gustavsson A., Hellman U., Sletten K., Murphy C. L., Weiss D. T., Solomon A., Olofsson B. O., and Westermark P. (2005) Amyloid deposits in transthyretin-derived amyloidosis: cleaved transthyretin is associated with distinct amyloid morphology. J. Pathol 206, 224–232 15810051 10.1002/path.1759

[46] Sievers S. A., Karanicolas J., Chang H. W., Zhao A., Jiang L., Zirafi O., Stevens J. T., Münch J., Baker D., and Eisenberg D. (2011) Structure-based design of non-natural amino-acid inhibitors of amyloid fibril formation. Nature 475, 96–100 21677644 10.1038/nature10154 PMC4073670

[47] Jiang L., Liu C., Leibly D., Landau M., Zhao M., Hughes M. P., and Eisenberg D. S. (2013) Structure-based discovery of fiber-binding compounds that reduce the cytotoxicity of amyloid β. Elife 2, e00857 23878726 10.7554/eLife.00857 PMC3713518

[48] Palaninathan S. K., Mohamedmohaideen N. N., Snee W. C., Kelly J. W., and Sacchettini J. C. (2008) Structural insight into pH-induced conformational changes within the native human transthyretin tetramer. J. Mol. Biol. 382, 1157–1167 18662699 10.1016/j.jmb.2008.07.029

[49] Bulawa C. E., Connelly S., Devit M., Wang L., Weigel C., Fleming J. A., Packman J., Powers E. T., Wiseman R. L., Foss T. R., Wilson I. A., Kelly J. W., and Labaudinière R. (2012) Tafamidis, a potent and selective transthyretin kinetic stabilizer that inhibits the amyloid cascade. Proc. Natl. Acad. Sci. U.S.A. 109, 9629–9634 22645360 10.1073/pnas.1121005109 PMC3386102

[50] Sekijima Y. (2014) Recent progress in the understanding and treatment of transthyretin amyloidosis. J. Clin. Pharm. Ther. 39, 225–233 24749898 10.1111/jcpt.12145

[51] Abedini A., and Raleigh D. P. (2006) Destabilization of human IAPP amyloid fibrils by proline mutations outside of the putative amyloidogenic domain: is there a critical amyloidogenic domain in human IAPP? J. Mol. Biol. 355, 274–281 16303136 10.1016/j.jmb.2005.10.052

[52] Chiu C. C., Singh S., and de Pablo J. J. (2013) Effect of proline mutations on the monomer conformations of amylin. Biophys. J. 105, 1227–1235 24010666 10.1016/j.bpj.2013.07.029 PMC3762349

[53] Colletier J. P., Laganowsky A., Landau M., Zhao M., Soriaga A. B., Goldschmidt L., Flot D., Cascio D., Sawaya M. R., and Eisenberg D. (2011) Molecular basis for amyloid-β polymorphism. Proc. Natl. Acad. Sci. U.S.A. 108, 16938–16943 21949245 10.1073/pnas.1112600108 PMC3193189

[54] Lim K. H., Dyson H. J., Kelly J. W., and Wright P. E. (2013) Localized structural fluctuations promote amyloidogenic conformations in transthyretin. J. Mol. Biol. 425, 977–988 23318953 10.1016/j.jmb.2013.01.008 PMC3594634

[55] Eldridge B., Cooley R. N., Odegrip R., McGregor D. P., Fitzgerald K. J., and Ullman C. G. (2009) An *in vitro* selection strategy for conferring protease resistance to ligand binding peptides. Protein Eng. Des. Sel. 22, 691–698 19755412 10.1093/protein/gzp052

[56] Takahashi T., and Mihara H. (2008) Peptide and protein mimetics inhibiting amyloid β-peptide aggregation. Acc. Chem. Res. 41, 1309–1318 18937396 10.1021/ar8000475

[57] Kroth H., Ansaloni A., Varisco Y., Jan A., Sreenivasachary N., Rezaei-Ghaleh N., Giriens V., Lohmann S., López-Deber M. P., Adolfsson O., Pihlgren M., Paganetti P., Froestl W., Nagel-Steger L., Willbold D., Schrader T., Zweckstetter M., Pfeifer A., Lashuel H. A., and Muhs A. (2012) Discovery and structure activity relationship of small molecule inhibitors of toxic β-amyloid-42 fibril formation. J. Biol. Chem. 287, 34786–34800 22891248 10.1074/jbc.M112.357665 PMC3464581

[58] Alves I. L., Divino C. M., Schussler G. C., Altland K., Almeida M. R., Palha J. A., Coelho T., Costa P. P., and Saraiva M. J. (1993) Thyroxine binding in a TTR Met 119 kindred. J. Clin. Endocrinol. Metab. 77, 484–488 8102146 10.1210/jcem.77.2.8102146

